# LncRNA MIR155HG contributes to smoke-related chronic obstructive pulmonary disease by targeting miR-128-5p/BRD4 axis

**DOI:** 10.1042/BSR20192567

**Published:** 2020-03-13

**Authors:** Jie Song, Qihu Wang, Liguo Zong

**Affiliations:** 1 Department of Respiratory Medicine, Yantai Yuhuangding Hospital, Yantai, Shandong, China; 2 Department of Respiratory Medicine, Muping Hospital of Traditional Chinese Medicine, Yantai, Shandong, China; 3 Department of Internal Medicine, Zaozhuang Municipal Hospital, Zaozhuang, Shandong, China

**Keywords:** BRD4, COPD, HPMECs, miR-128-5p, MIR155HG

## Abstract

Chronic obstructive pulmonary disease (COPD) is a common airway disease characterized by an exaggerated pulmonary inflammatory response. Long noncoding MIR155 host gene (lncRNA MIR155HG) has been identified to be related to the macrophage polarization in COPD. However, the detailed function of MIR155HG in cigarette smoke (CS)-mediated COPD remains largely unknown. The expression level of MIR155HG was elevated while miR-218-5p was decreased in lung tissues of smokers without or with COPD, especially in smokers with COPD, and cigarette smoke extract (CSE)-treated human pulmonary microvascular endothelial cell (HPMECs) in a dose- and time-dependent manner. Then, functional experiments showed that MIR155HG deletion could reverse CSE exposure-induced apoptosis and inflammation in HPMECs. MiR-218-5p was confirmed to be a target of MIR155HG and rescue assay showed miR-218-5p inhibitor attenuated the inhibitory action of MIR155HG knockdown on CSE-induced HPMECs. Subsequently, miR-218-5p was found to target bromodomain containing 4 (BRD4) directly, and miR-218-5p overexpression overturned CSE-induced injury of HPMECs via regulating BRD4. Additionally, co-expression analysis indicated MIR155HG indirectly regulated BRD4 expression in HPMECs via miR-218-5p. Thus, we concluded that MIR155HG contributed to the apoptosis and inflammation of HPMECs in smoke-related COPD by regulating miR-128-5p/BRD4 axis, providing a novel insight on the pathogenesis of COPD and a therapeutic strategy on COPD treatments.

## Introduction

Chronic obstructive pulmonary disease (COPD), a common airway disease, is characterized by chronic bronchiolitis and emphysema induced by the exaggerated pulmonary inflammatory response that was triggered by the interactions of genetic and environmental risk factors, and of which cigarette smoking (CS) is recognized as the best-defined risk factor and response to about 80–90% of the COPD cases [1,2]. CS can initiate pulmonary vascular impairment via directly injuring endothelial cells, then result in the enhancement of cell apoptosis, reduced epithelial barrier formation and high levels of oxidative stress, ultimately leading to a sustained inflammatory response in the lung tissue [3–5]. However, the molecular mechanisms underlying the CS-induced apoptosis and inflammation in the development of COPD are still largely unclear.

Long noncoding RNAs are a class of ncRNAs with longer than 200 nucleotides in size, which can modulate the expression of protein-coding genes by cis- or trans-regulatory effects [6]. LncRNAs have been identified to implicate in various physiological and pathological processes and to be dysregulated in various human diseases [7]. MicroRNA 155 host gene (MIR155HG), a novel identified lncRNA, encodes the microRNA (miR)-155, which regulates multiple signaling pathways of innate and adaptive immune responses against viral infections [8]. Additionally, MIR155HG has been revealed to function as an oncogene in the progression of diverse cancers, such as pancreatic cancer, glioblastoma and laryngeal squamous cell carcinoma [9–11]. In recent, Li et al*.* found MIR155HG regulated M1/M2 macrophage polarization in COPD through regulating interleukins (IL)- 1β, IL-10, IL-12 and tumor necrosis factor-α (TNF-α) expression, suggesting that MIR155HG might involve in the development of COPD [12]. However, the exact function of MIR155HG in COPD pathogenesis remains elusive.

MicroRNAs (miRNAs) belong to a class of conserved, small endogenous non-coding RNAs that negatively regulate gene expression at the post-transcriptional level through complementary binding to the 3′-untranslated region (3′-UTR) of their target mRNAs, leading to the suppression of protein synthesis and cleavage of mRNAs [13,14]. Up to date, an increasing number of evidence demonstrated that miRNAs play important roles in the pathogenesis of COPD [15]. MiR-218-5p, a member of the miR-218 family, has been investigated to be implicated in various human malignancies [16,17]. Recently, emerging evidence suggested that miR-218-5p participated in the pathogenesis of COPD [18], suggesting the regulatory role of miR-218-5p in the progression of COPD. Bromodomain protein 4 (BRD4), a member of the Bromodomain and Extra-Terminal domain (BET) protein family, plays an vital role in the process of gene transcription [19], which can directly and indirectly modulate transcription both as a passive scaffold through recruiting vital transcription factors and as an active kinase that phosphorylates RNA polymerase [19]. Previous studies have shown that BRD4 suppression significantly decreases the expression levels of pro-inflammatory cytokines both *in vivo* and *in vitro* [20,21], indicating BRD4 implicate in the inflammatory process.

In the present study, we explored the expression patterns of MIR155HG in lung tissues of smokers without or with COPD and HPMECs, identified the biological function of MIR155HG on HPMECs treated with cigarette smoke extract (CSE). In addition, we also investigated the regulatory relationship among MIR155HG, miR-218-5p and BRD4 in the progression of COPD.

## Materials and methods

### Patients and specimens

Lung specimens were collected from 49 patients who underwent pneumonectomy for a solitary non-small cell lung cancer (at least 5-cm away from the lesion) at Yantai Yu huang ding Hospital. Patient information, including age, sex, smoking history, body mass index (BMI), lung function test results (forced vital capacity (FVC), forced expiratory volume in one-second (FEV_1_), FEV_1_ (% predicted), FEV_1_/FVC) and other contaminants, was listed in Table 1. The samples were divided into three groups: non-smokers without COPD (*n* = 11); smokers without COPD (*n* = 17) and smokers with COPD (*n* = 21). A person who never smoked or smoked fewer than 100 cigarettes in his lifetime was considered as a never smoker. Smokers included those presently smoking and those who quit smoking 12 months before the interview. COPD was diagnosed in accordance with the criteria of the Global Initiative for Chronic Obstruct Lung Disease (GOLD). Recruiting COPD patients were severe COPD (GOLD III and IV classified according to spirometric data).

**Table 1:** Characteristics of subjects in the present study

	Non-smokers	Smokers	Smokers with COPD
Number	11	17	21
Age (years)	63.8 ± 5.2	65.2 ± 6.3	66.8 ± 7.1
Male	11(100%)	17(100%)	21(100%)
Smoking history (pack-years)	0	40.3 ± 4.7**	50.5 ± 5.2**^#^
BMI (kg/m^2^)	25.8 ± 3.2	29.1 ± 5.3	27.2 ± 6.3
FEV_1_ (L)	3.25 ± 0.12	2.98 ± 0.18	2.11 ± 0.15*^#^
FVC (L)	4.06 ± 0.23	3.61 ± 0.25	3.52 ± 0.18
FEV_1_/FVC%	80.05 ± 1.6	82.55 ± 1.8	64.92 ± 2.1**^##^
FEV_1_(% predicted)	105.4 ± 5.3	98.6 ± 4.6	68.3 ± 3.9**^##^
Occupational dust exposure, *n*			
Yes	2	1	2
No	9	16	19
Occupational hazard gas exposure, *n*			
Yes	1	2	2
No	10	15	19

Abbreviations: COPD: Chronic Obstructive Pulmonary Disease; BMI: body mass index; FVC: forced vital capacity; FEV_1_ : forced expiratory volume in one second. *P <0.05, ** *P* <0.01, different from non-smoker. # *P* <0.05, ## *P* <0.01 different from smokers.

All specimens were clinically stable without any chemotherapy or radiotherapy treatment. COPD patients had only received bronchodilators and none of them had received any corticosteroids or antibiotics 3 months before resection; besides, patients with comorbidities, including asthma, pulmonary infection, a history of other respiratory diseases, heart failure, and/or neuromuscular disease, were excluded. All participants involved in the present study have provided written informed consent and the study protocols were approved by the Ethics Committee of Yantai Yu huang ding Hospital.

### Cigarette Smoke Extract (CSE) preparation

Cigarette smoke extract (CSE) was prepared by a modification of the method reported previously [22]. In brief, one commercial cigarette was combusted with a modified syringe-driven apparatus. The smoke was bubbled through 25 ml of media over 5 min by drawing 35 ml smoke every 15 s. The resulting suspension was filtered through a 0.2 μm pore-size filter to remove large particles and bacteria. This 100% CSE sample was serially diluted to working concentrations of 0.5%, 1%, 2.5%, and 5% with PBS, and used within 30 min for the following experiments. For the negative control (NC), cells were treated with normal medium without any CSE.

### Cell culture and treatment

HPMECs were obtained from Shanghai Cell Bank of the Chinese Academy of Science (Shanghai, China) and were cultured in the endothelial cell medium (Gibco, Carlsbad, CA, U.S.A.) containing with 10% heated-inactivated fetal bovine serum (FBS, Gibco), 1% endothelial cell growth factor (Merck, Darmstadt, Germany), 100 IU/ml penicillin (Gibco), and 50 μg/ml streptomycin (Gibco). Then, cells were incubated in a humidified incubator at 37°Cwith 5% CO_2_.

MiR-218-5p mimic (miR-218-5p), mimic negative control (miR-NC), miR-218-5p inhibitor (in-miR-218-5p) and inhibitor negative control (in-miR-NC) were purchased from RIBOBIO (Guangzhou, China), pcDNA3.1-MIR155HG overexpression vector (MIR155HG), pcDNA3.1-BRD4 overexpression vector (BRD4), pcDNA3.1 empty vector (pcDNA), small interfering RNA (siRNA) against MIR155HG (si-MIR155HG) or siRNA negative control (si-NC) were obtained from Genepharma (Shanghai, China). All the oligonucleotides or vectors were transfected into HPMECs cells using Lipofectamine 2000 (Invitrogen, Carlsbad, CA, U.S.A.) according to the manufacturer’s instructions. After transfection for 48h, cells were harvested for subsequent analysis.

### Quantitative real-time polymerase chain reaction

Total RNA was isolated from cells or tissues with the help of Trizol reagent (Invitrogen) according to the manufacturer’s protocol. To detect the expression levels of MIR155HG and BRD4, cDNA was reversed from 2 μg of total RNA using a PrimeScript RT Master Mix Kit (Takara, Shiga, Japan). For detecting miR-218-5p level, cDNA was synthesized using miScript II RT kit (Takara). Then, quantitative PCR was performed using SYBR Green PCR Master Mix kit (Takara) on a PRISM® 7300 system (Applied Biosystems, Foster City, CA, U.S.A.). Glyceraldehyde-3-phosphate dehydrogenase (GAPDH) or U6 was used as an internal inference and the relative expression level was evaluated by 2^−ΔΔCt^ method. The special primers were listed: MIR155HG, forward 5′-CCCAAATCTAGGTTCAAGTTC-3′, reverse 5′-ATCTAAGCCTCACAACAAC-3′; GAPDH, forward 5′-AGGTGAAGGTCGGAGTCAACG-3′, reverse 5′-AGGGGTCATTGATGGCAACA-3; BRD4, forward 5′-CCCCTCGTGGTGGTGAAG-3′, reverse 5′-GCTCGCTGCGGATGATG-3′; miR-218-5p, forward 5′-AACACGAACTAGATTGGTACA-3′, reverse 5′-AGTCTCAGGGTCCGAGGTATTC-3′; U6, forward 5′-GCTTCGGCAGCACATATACTAAAAT-3′, reverse 5′-CGCTTCACGAATTTGCGTGTCAT-3′.

### Cell apoptosis

Cell apoptosis was examined by the flow cytometry using Annexin V-FITC/PI apoptosis detection kit (Solarbio, Beijing, China). Briefly, 5 × 10^5^ cells were resuspended in binding buffer at the specific time points, and then stained with 5 μl Annexin V-FITC and PI for 10 min in the dark. Finally, cell apoptosis rate was analyzed by flow cytometry (BD Biosciences, San Jose, CA, U.S.A.).

### Western blot

Following the transfection or treatment of CSE, cells were harvested and lysed in RIPA lysis buffer (Beyotime, Shanghai, China). Then, total protein was quantified using a bicinchoninic acid (BCA) protein assay kit (Beyotime) following the manufacturer’s instructions. Equal amounts of protein were separated on SDS-polyacrylamide gels and transferred onto polyvinylidene difluoride membranes (Millipore, Billerica, MA, U.S.A.). After blocking with 5% non-fat milk for 1 h at room temperature, the membranes were incubated with primary antibodies against BRD4, B-cell lymphoma (Bcl-2), Bcl-2 associated X (Bax) and Cleaved-caspase3 as well as β-actin overnight at 4°C, and then interacted with secondary antibody (HRP-conjugated anti-rabbit IgG) for 2 h at room temperature. Immunoreactive signals were visualized through commercial enhanced chemiluminescence chromogenic substrate (Beyotime) and quantitated by Image Lab software (Bio-Rad, Hercules, CA, U.S.A.).

### ELISA analysis

Cytokine concentrations from the supernatants of HPMECs after appropriate treatment were measured by using commercial IL-6, IL-8 and TNF-α ELISA kits (R&D Systems, Minneapolis, U.S.A.) in accordance with the manufacturer’s instructions.

### Luciferase reporter assay

The MIR155HG or BRD4 3′-UTR containing wild-type (WT) or mutant (MUT) binding sequences of miR-218-5p was cloned into the pGL3-reporter plasmid (Promega, Shanghai, China), respectively. Then, cells were cultured in 96-well plates and co-transfected with miR-218-5p mimic or miR-NC mimic, corresponding luciferase reporters, or the control luciferase plasmid using Lipofectamine 2000 (Invitrogen). After transfection for 48 h, a dual luciferase assay kit (Promega) was used to investigate the luciferase activity following the manufacturer’s instructions.

### RNA immunoprecipitation (RIP)

RNA immunoprecipitation (RIP) assay was conducted by using Magna RNA immunoprecipitation kit (Millipore). HPMECs were transfected with miR-218-5p or miR-NC. After 48 h transfection, cells were lysed in RIP buffer harboring magnetic beads conjugated with AgO_2_ or IgG antibody. Subsequently, the immunoprecipitated RNAs were isolated by TRIzol reagent and enrichment of MIR155HG was analyzed by qRT-PCR.

### Statistical analysis

Data were presented as the mean ±standard deviation (S.D.). The differences between different groups were analyzed with Student’s *t* test or one-way analysis of variance (ANOVA) followed by Tukey post-hoc test on GraphPad Prism 7 (GraphPad Inc., San Diego, CA, U.S.A.). *P* <0.05 indicated statistically significant (*P <0.05, **P <0.01, ***P <0.001, ****P <0.0001). Each experiment was performed three times independently.

## Results

### Baseline characteristics

As presented in Table 1, no difference was found in age, gender or BMI among non-smokers, smokers and smokers with COPD groups (*P* >0.05). The pack-years of smokers with COPD were higher than that in smokers or non-smokers, respectively (*P* <0.05). Pulmonary function values FEV1, FEV1/FVC and FEV1% predicted were significantly lower in smokers with COPD than those in the smokers and non-smokers (*P* <0.05), while there was no significant difference in FVC among the three groups (*P* >0.05). Among those subjects, the occupational exposure to dust and toxic gas also did not differ significantly (*P* >0.05).

### MIR155HG is up-regulated but miR-218-5p is down-regulated in COPD tissues and CSE-mediated HPMECs

**Figure 1:**
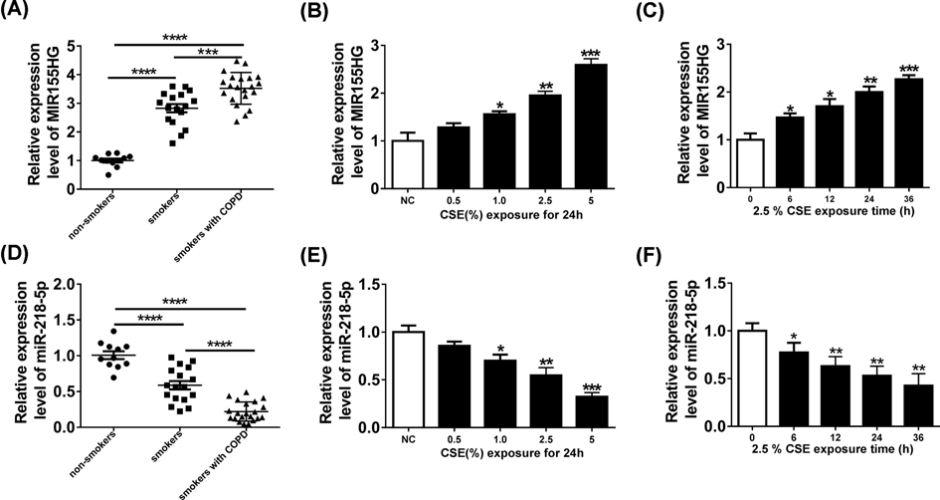
MIR155HG is up-regulated but miR-218-5p is down-regulated in COPD tissues and CSE-mediated HPMECs (**A** and **D**) MIR155HG and miR-218-5p levels in lungs from non-smokers, smokers, and smokers with COPD groups were detected using qRT-PCR. (**B** and** E**) The expression levels of MIR155HG and miR-218-5p in HPMECs 24 h following CSE exposure (0, 0.5, 1, 2, 2.5, 5%). (**C** and** F**) MIR155HG and miR-218-5p expression in HPMECs exposed to CSE for 0, 6, 12, 24 and 36 h; *P <0.05, **P< 0.01, ***P <0.001, ****P <0.0001.

The expression of MIR155HG in 49 cases of lung tissue specimens was measured using qRT-PCR and we found that compared with samples from non-smoker without COPD group, the expression level of MIR155HG was significantly elevated in lung tissues of smokers without or with COPD, especially in smokers with COPD group (Figure 1A). In addition, after exposure to a varying concentration of CSE (0, 0.5, 1, 2, 2.5, 5%) for 24 h, the expression of MIR155HG in HPMECs was discovered to be increased in a dose-dependent manner (Figure 1B). Furthermore, the expression of MIR155HG was also time-dependently up-regulated in HPMECs by 2.5% CSE exposure at 0, 6, 12, 24 and 36 h (Figure 1C). In addition, the expression of miR-218-5p was also detected in 49 cases of lung tissue specimens and a remarkably decreased expression of miR-218-5p in lung tissues of smokers without or with COPD was determined (Figure 1D). Moreover, the expression of miR-218-5p was also demonstrated to be decreased in a dose- and time-dependent manner in HPMECs (Figure 1E,F). Therefore, these findings indicated that aberrant expression of MIR155HG and miR-218-5p might be related to the pathogenesis of COPD.

### MIR155HG deletion suppresses CSE-induced apoptosis and inflammation in HPMECs

**Figure 2:**
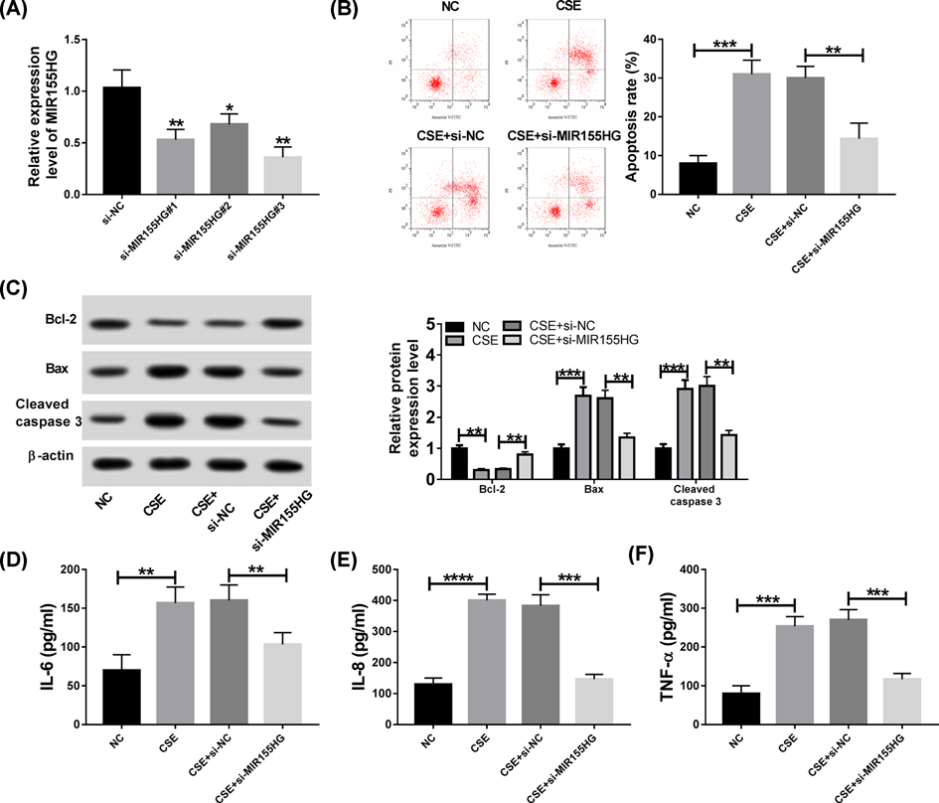
MIR155HG deletion suppresses CSE-induced apoptosis and inflammation in HPMECs HPMECs were transfected with si-NC or si-MIR155HG before treatment with 2.5% CSE. (**A**) The expression of MIR155HG was detected by qRT-PCR in HPMECs. (**B**) Cell apoptosis was measured by flow cytometry in HPMECs. (**C**) The protein expression levels of Bax, Bcl-2 and cleaved-caspase3 were measured by Western blot in HPMECs. (**D**–**F**) The levels of inflammatory cytokines IL-6, IL-8 and TNF-α were detected using ELISA assay in HPMECs; *P <0.05, **P <0.01, ***P <0.001, ****P <0.0001.

To explore the potential biological functions of MIR155HG in CSE-induced apoptosis and inflammation, HPMECs exposed with 2.5% CSE were transfected with si-NC or si-MIR155HG, and then the transfection efficiency was demonstrated using qRT-PCR with the results of decreased MIR155HG expression in HPMECs transfected with si-MIR155HG (Figure 2A). Subsequently, flow cytometry results showed CSE treatment markedly increased the apoptosis, while silencing of MIR155HG could significantly alleviate CSE-induced apoptosis of HPMECs (Figure 2B). Moreover, Western blot indicated that the increase of Bax and cleaved-caspase3 protein and decrease of Bcl-2 protein induced by 2.5% CSE exposure in HPMECs was attenuated by si-MIR155HG transfection in HPMECs (Figure 2C), further validating that MIR155HG deletion suppressed CSE-induced apoptosis. Additionally, the secretion levels of inflammatory cytokines IL-6, IL-8 and TNF-α in HPMECs were determined. Results showed 2.5% CSE exposure significantly stimulated the expression levels of IL-6, IL-8 and TNF-α compared with control cells with air exposure in HPMECs, while these effects could be inhibited by MIR155HG silencing (Figure 2D–F). In all, we illustrated that MIR155HG deletion could suppress CSE-induced apoptosis and inflammation in HPMECs.

### MIR155HG is a sponge of miR-218-5p and negatively regulates its expression in CSE-treated HPMECs

**Figure 3:**
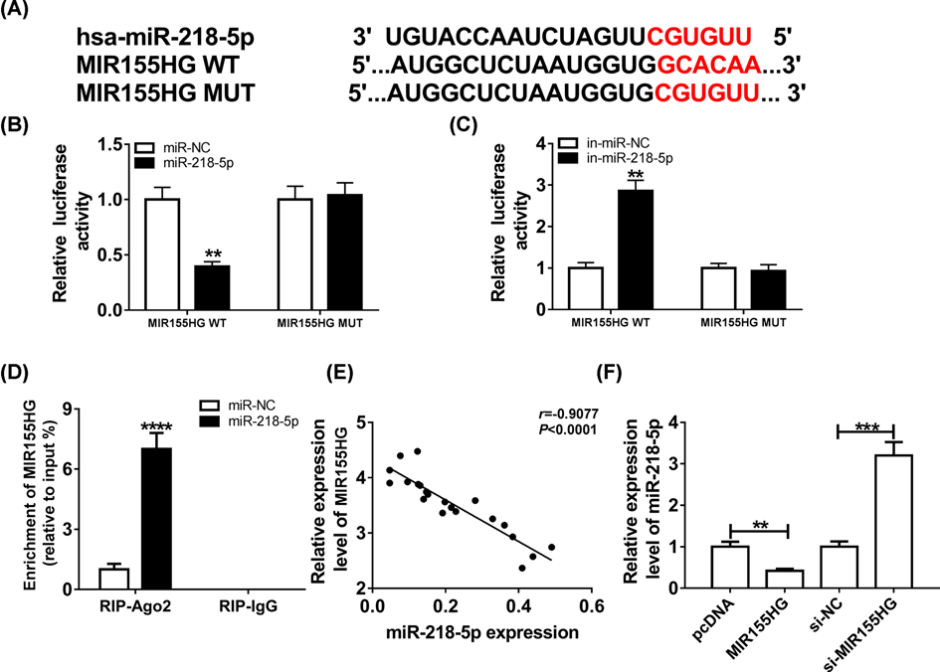
MIR155HG is a sponge of miR-218-5p and negatively regulates its expression in CSE-treated HPMECs (**A**) The potential binding sites of miR-218-5p in the MIR155HG were predicted by bioinformatics analysis. (**B** and **C**) The Luciferase activity was measured in HPMECs co-transfected with MIR155HG-WT or MIR155HG-MUT and miR-NC, miR-218-5p or in-miR-NC, in-miR-218-5p. (**D**) The enrichment of MIR155HG was examined in HPMECs transfected with miR-NC or miR-218-5p via RIP. (**E**) Correlation analysis between miR-218-5p and MIR155HG expression. (**F**) The expression of miR-218-5p was detected using qRT-PCR in HPMECs transfected with MIR155HG or si-MIR155HG; **P <0.01, ***P <0.001, ****P <0.0001.

According to the prediction of DIANA-LncBaseV2 prediction program, miR-218-5p might be a potential target of MIR155HG (Figure 3A). To verify this prediction, luciferase reporter assay was performed and we found miR-218-5p mimic transfection reduced the luciferase activity of the MIR155HG-WT reporter vector but not MIR155HG-MUT reporter vector in HPMECs (Figure 3B). However, miR-218-5p inhibitor transfection enhanced the luciferase activity of the MIR155HG-WT reporter vector and there was no obvious change in MIR155HG -MUT reporter after reduction of miR-218-5p in HPMECs (Figure 3C). Meanwhile, RIP assay further confirmed the direct interaction between miR-218-5p and MIR155HG because of significant enrichment of MIR155HG in HPMECs (Figure 3D). Additionally, a negatively between MIR155HG and miR-218-5p in the tissues of smokers with COPD was observed (Figure 3E), and MIR155HG overexpression inhibited miR-218-5p expression, while MIR155HG deletion promoted miR-218-5p expression in HPMECs (Figure 3F). Thus, MIR155HG was a sponge of miR-218-5p and negatively regulated its expression.

### MIR155HG deletion suppresses CSE-induced apoptosis and inflammation in HPMECs via regulating miR-218-5p

**Figure 4:**
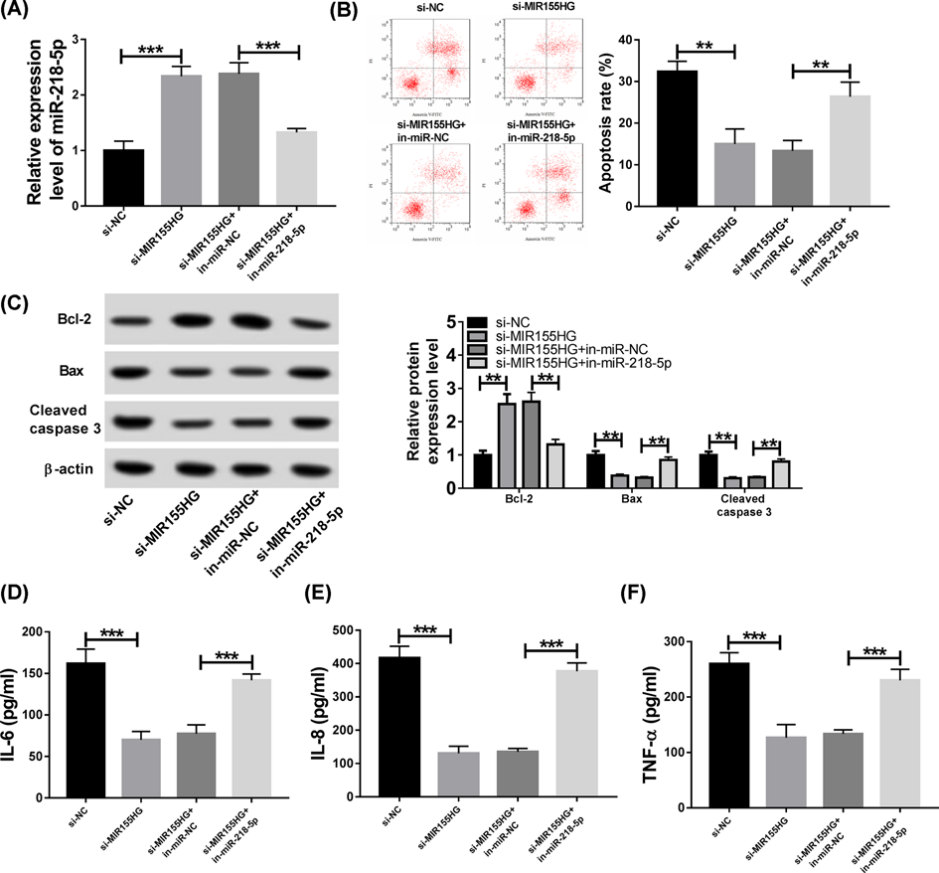
MIR155HG deletion suppresses CSE-induced apoptosis and inflammation in HPMECs via regulating miR-218-5p HPMECs were transfected with si-NC, si-MIR155HG, si-MIR155HG + in-miR-NC or si-MIR155HG + in-miR-218-5p before treatment with 2.5% CSE. (**A**) The expression of miR-218-5p was detected in HPMECs. (**B**) Cell apoptosis was measured by flow cytometry in HPMECs. (**C**) The protein expression levels of Bax, Bcl-2 and cleaved-caspase3 were measured by Western blot in HPMECs. (**D**–**F**) The levels of inflammatory cytokines IL-6, IL-8 and TNF-α were detected using ELISA assay in HPMECs; **P <0.01, ***P <0.001.

Whether miR-218-5p involved in the activity of MIR155HG on CSE-induced apoptosis and inflammation was further investigated, HPMECs were transfected with si-NC, si-MIR155HG, si-MIR155HG + in-miR-NC or si-MIR155HG + in-miR-218-5p before treatment with 2.5% CSE, as expected, miR-218-5p inhibition reversed MIR155HG knockdown mediated up-regulation of miR-218-5p, suggesting successful transfection (Figure 4A). After exposure with 2.5% CSE, cell apoptosis and inflammation were determined and results showed that miR-218-5p inhibitor transfection reversed MIR155HG deletion-induced suppression on CSE-induced apoptosis (Figure 4B,C) and inflammation (Figure 4D–F) in HPMECs. Taken together, MIR155HG contributed to CSE-induced apoptosis and inflammation in HPMECs via regulating miR-218-5p.

### MiR-218-5p directly targets BRD4 and suppresses its expression in CSE-treated HPMECs

**Figure 5:**
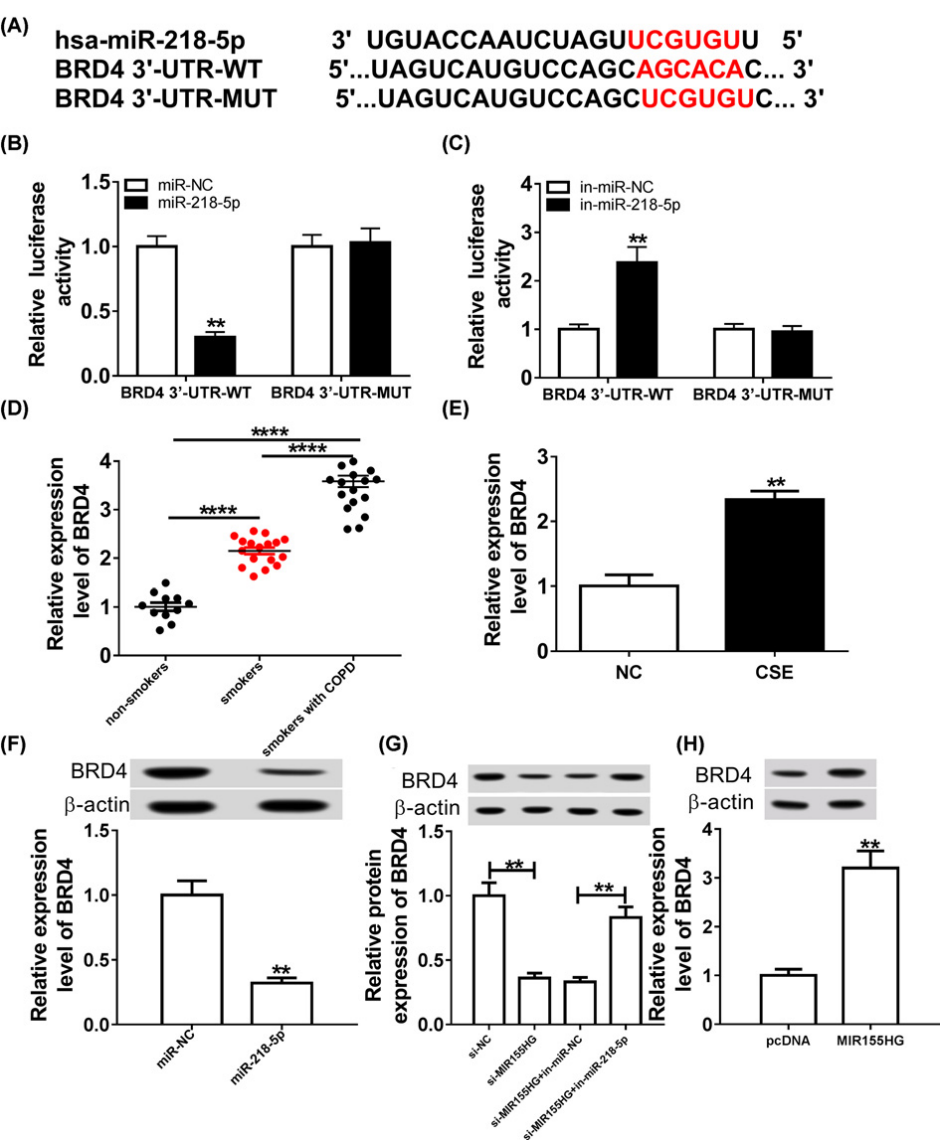
MiR-218-5p directly targets BRD4 and suppresses its expression in CSE-treated HPMECs (**A**) The potential binding sites between miR-218-5p and BRD4 were listed. (**B** and**C**) The Luciferase activity was analyzed in HPMECs co-transfected with BRD4-WT or BRD4-MUT and miR-NC, miR-218-5p or in-miR-NC, in-miR-218-5p. (**D**) TheBRD4 expression level in lungs from non-smokers, smokers, and smokers with COPD groups was detected using qRT-PCR. (**E**) The expression level of BRD4 in HPMECs 24 h following CSE exposure (2.5%) was examined. (**F**) The expression of BRD4 was demonstrated using Western blot in HPMECs transfected with miR-NC or miR-218-5p. (**G**) The level of BRD4 protein was determined using Western blot in HPMECs transfected with si-NC, si-MIR155HG, si-MIR155HG and in-miR-NC or si-MIR155HG and in-miR-218-5p. (**H**) The protein expression of BRD4 in HPMECs transfected with pcDNA or BRD4 using Western blot; **P <0.01.

The potential target gene of miR-218-5p was predicted using DianaTools program, and BRD4 was found that might be a target of miR-218-5p (Figure 5A). Then luciferase reporter analysis exhibited that the relative luciferase activity of the BRD4-WT reporter vector was dramatically inhibited by miR-218-5p overexpression, whereas was increased by miR-218-5p inhibition in HPMECs, meanwhile, no significance was observed in BRD4-MUT reporter vector co-transfection groups (Figure 5B,C). All these data suggested the direct interaction of miR-218-5p and BRD4. Subsequently, the expression of BRD4 was detected and we discovered BRD4 expression was significantly up-regulated in lung tissues of smokers without or with COPD, especially in smokers with COPD group compared with samples from non-smoker without COPD group (Figure 5D). Besides that, CSE exposure significantly stimulated BRD4 expression in HPMECs (Figure 5E). Additionally, we also found BRD4 was repressed by miR-218-5p overexpression (Figure 5F). Thus, miR-218-5p targetedly suppressed BRD4. Moreover, we further studied whether MIR155HG regulated BRD4 via miR-218-5p, results showed BRD4 expression was increased by MIR155HG overexpression, and was decreased by MIR155HG depletion in HPMECs (Figure 5G,H); importantly, miR-218-5p inhibition rescued the level of BRD4 reduced by MIR155HG depletion (Figure 5G). Altogether, MIR155HG positively regulated BRD4 via miR-218-5p in HPMECs.

### Overexpressed miR-218-5p inhibits CSE-induced apoptosis and inflammation in HPMECs via regulating BRD4

**Figure 6:**
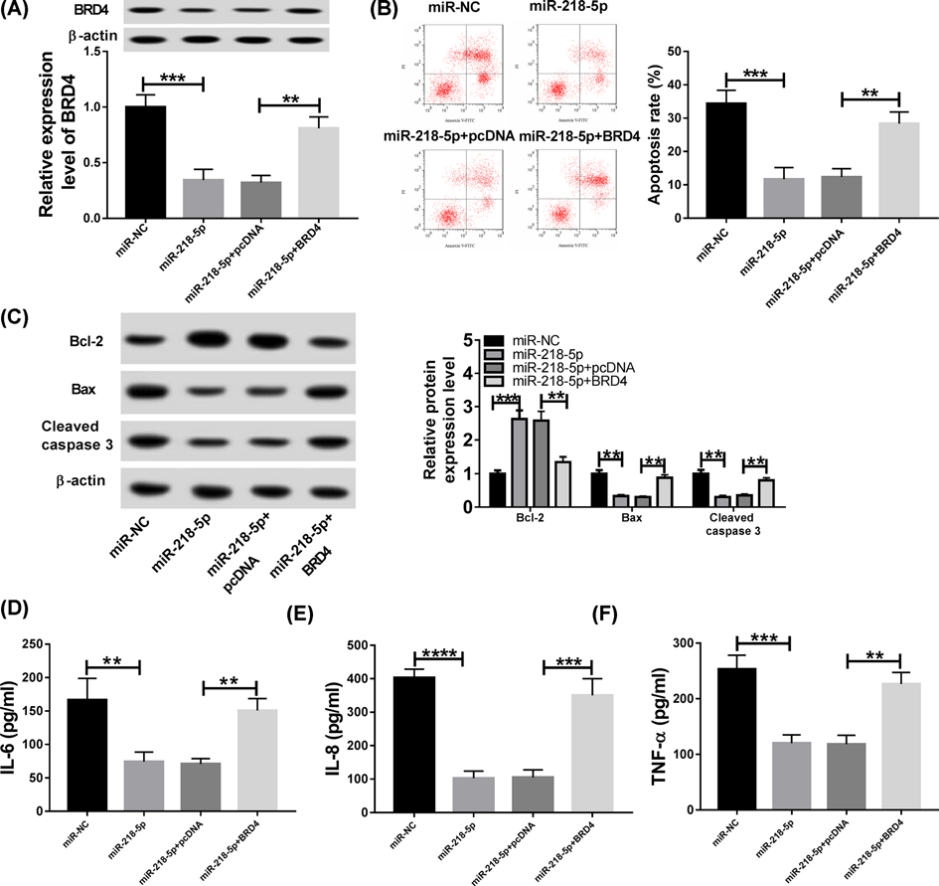
Overexpressed miR-218-5p inhibits CSE-induced apoptosis and inflammation in HPMECs via regulating BRD4 HPMECs were transfected with miR-NC or miR-218-5p or miR-218-5p + pcDNA or miR-218-5p + BRD4 prior to exposure with 2.5% CSE. (**A**) The expression of BRD4 was detected by Western blot in HPMECs. (**B**) Cell apoptosis was measured by flow cytometry in HPMECs. (**C**) The protein expression levels of Bax, Bcl-2 and cleaved-caspase3 were measured by Western blot in HPMECs. (**D**–**F**) The levels of inflammatory cytokines IL-6, IL-8 and TNF-α were detected using ELISA assay in HPMECs; *P<0.05, **P<0.01, ***P<0.001, ****P<0.0001.

To further investigate the underlying molecular mechanism by which miR-218-5p modulating the apoptosis and inflammation in CSE-treated HPMECs. HPMECs were transfected with miR-NC, miR-218-5p, miR-218-5p + pcDNA, or miR-218-5p + BRD4 prior to exposure with 2.5% CSE, and BRD4 transfection restored miR-218-5p overexpression-mediated inhibition on BRD4 levels as expected (Figure 6A). Then cells were exposed to 2.5% CSE to detect cell apoptosis and inflammation. Results exhibited overexpressed miR-218-5p notably suppressed cell apoptosis, reflected by the decreased apoptosis rate, Bax and cleaved caspase-3 levels, as well as the increase of anti-apoptosis protein Bcl-2 expression in CSE-induced HPMECs, while BRD4 vector transfection could attenuate these effects (Figure 6B,C). Afterward, ELISA assay indicated the increase of inflammatory cytokines IL-6, IL-8 and TNF-α levels induced by miR-218-5p mimic transfection was significantly abated by overexpressed BRD4 in CSE-induced HPMECs (Figure 6D–F). These results suggested that miR-218-5p overexpression inhibited CSE-induced apoptosis and inflammation in HPMECs via regulating BRD4.

## Discussion

COPD is characterized by partly reversible airflow obstruction, inflammation in the airways and other systemic effects [23]. Over the past decades, emerging evidence has reported that lncRNAs may involve in the pathogenesis of COPD. LncRNAs expression profile in lung tissue from non-smokers and smokers without or with COPD has also been published [24]. However, only a few pieces of research have focused on the mechanisms of lncRNAs in epithelial responses or that investigate their effects on modulating inflammatory responses in COPD. For instance, Zheng et al*.* illustrated that lncRNA COPDA1 promotes the proliferation of HBSMCs and may involve in facilitating airway remodeling in COPD [25]. Tang et al*.* found knockdown of lncRNA TUG1 promoted cell proliferation after TGF-β treatment through inhibiting the expression levels of α-SMA and fibronectin in COPD [26]. The smoke and cancer-associated lncRNA-1 (SCAL1) was found to be up-regulated by CSE *in vitro* and mediated some of the cytoprotective functions of nuclear factor erythroid 2 related factor (NRF2) toward CS-induced stress in lung cancer cells [27].

In the present study, we found MIR155HG was elevated in lung tissues of smokers without or with COPD, especially in smokers with COPD, indicating the potential regulatory role of MIR155HG in COPD. CS that can initiate apoptosis in macrophages, fibroblasts, vascular endothelial cells and alveolar epithelial cell lines [28–30] is considered as the best-defined risk factor for COPD cases and can contribute to irreversible pathological changes in lung tissue, including airway remodeling and pulmonary emphysema [31]. In the present study, CSE treated promoted the expression of MIR155HG in HPMECs in a dose- and time-dependent manner. Besides that, 2.5% CSE exposure could apparently induce apoptosis, reflected by the increased apoptosis rate and Bax and cleaved caspase-3 level, as well as the decreased expression of anti-apoptosis protein Bcl-2, and the expression levels of IL-6, IL-8 and TNF-α compared with control cells with air exposure in HPMECs. Afterward, to explore the biological function of MIR155HG on COPD progression, loss-of-function assay was performed and we demonstrated that MIR155HG deletion could reverse CSE exposure induced apoptosis and inflammation in HPMECs.

To investigate the molecular mechanism underlying these actions, the possible targets regulated by MIR155HG was predicated and confirmed using bioinformatics analysis. Then, we identified that miR-218-5p was a target of MIR155HG. In previous studies, Conickx et al*.* reported that miR-218-5p was decreased in CS-exposed mice and human bronchial epithelial cells (HBECs) and strongly correlated with airway obstruction, besides, miR-218-5p repressed the CS-induced increase in inflammatory cells and chemokine (CCL20 and IL-8) *in vitro* and *in vivo* [32]*.* Xu et al*.* revealed that miR-218 was involved in Mucin 5AC hyper-production and inflammation by repressing TNFR1-mediated activation of NF-κB in smoking-induced HBECs of COPD [33]. In addition, Song et al*.* demonstrated that miR-218-5p showed a protective role in CS-induced inflammation in CS-induced COPD mice [18]. In the present study, miR-218-5p was decreased in lung tissues of smokers without or with COPD, and in CSE-treated HPMECs. Subsequently, functional analysis showed that overexpressed miR-218-5p notably suppressed cell apoptosis and the levels of inflammatory cytokines IL-6, IL-8 and TNF-α, indicating the protective effects of miR-218-5p on CSE-induced HPMECs apoptosis and inflammation in COPD, which was consistent with the previous researches. Afterward, rescue assay was performed and we discovered that miR-218-5p inhibitor transfection could reverse MIR155HG deletion induced suppression on CSE-induced injury in HPMECs, suggesting MIR155HG contributed to CSE-induced apoptosis and inflammation in HPMECs via regulating miR-218-5p.

BRD4 has been revealed to play a vital role in the regulation of inflammatory genes. Khan et al*.* found Brd4 inhibition could significantly reduce oxidative stress enhanced IL1β-induced IL-6 and CXCL8 expression in human airway epithelial cells [34]. Pan-BET protein, including BRD4, regulates the expression of genes involved in T cell regulation and innate pathways in COPD patients [35]. Additionally, Tang et al*.* investigated that miR-29b might participate in the airway inflammation in COPD by regulating inflammatory cytokine expression through targeting BRD4 [36]. All the evidence indicated the important role of BRD4 in the inflammation of COPD. In the present study, BRD4 was predicted and confirmed to be a target of miR-218-5p and rescue assay analysis showed miR-218-5p exerted inhibitory effects on CSE-induced apoptosis and inflammation in HPMECs via regulating BRD4. Additionally, co-expression analysis indicated MIR155HG positively regulate BRD4 expression in HPMECs via miR-218-5p.

Although some interesting results were found in the present study, there were still some limitations. First, the sample size was relatively small in the present study, which might cause poor statistic power. Second, all subjects were collected from our hospital. Third, the correlation between lung function values, such as FEV1/FVC%, and expression of MIR155HG, miR-218-5p and BRD4 was not investigated. Therefore, further study with more COPD patients from multicenter is greatly necessary.

In sum up, our results indicated that MIR155HG was elevated in lung tissues of smokers without or with COPD and CSE-induced HPMECs. MIR155HG contributed to apoptosis and inflammation of HPMECs in smoke-related COPD by regulating miR-128-5p/BRD4 axis, which provided novel insight into the pathogenesis of COPD and therapeutic strategies on COPD treatments.

## Abbreviations

3′-UTR: 3′-untranslated region
BCA: bicinchoninic acid
BET: bromodomain and extra-terminal domain
BRD4: bromodomain protein 4
COPD: chronic obstructive pulmonary disease
CS: cigarette smoke
CSE: cigarette smoke extract
FBS: fetal bovine serum
GAPDH: glyceraldehyde-3-phosphate dehydrogenase
GOLD: Global Initiative for Chronic Obstruct Lung Disease
HBEC: human bronchial epithelial cell
HPMEC: human pulmonary microvascular endothelial cell
IL: interleukin
in-miR-218-5p: miR-218-5p inhibitor
MIR155HG: MicroRNA 155 host gene
MIR155HG: MIR155 host gene
MUT: mutant
RIP: RNA immunoprecipitation
S.D.: standard deviation
siRNA: small interfering RNA
TNF-α: tumor necrosis factor-α
WT: wild-type
