# Expression of Concern: LncRNA MIR155HG contributes to smoke-related chronic obstructive pulmonary disease by targeting miR-128-5p/BRD4 axis

**DOI:** 10.1042/BSR-2019-2567_EOC

**Published:** 2024-10-07

**Keywords:** HPMECs, miR-128-5p, MIR155HG

The Editorial Office has been made aware of potential issues surrounding the scientific validity of this paper “LncRNA MIR155HG contributes to smoke-related chronic obstructive pulmonary disease by targeting miR-128-5p/BRD4 axis” DOI: 10.1042/BSR20192567, hence has issued an expression of concern to notify readers whilst the Editorial Office investigates. It has been noted that the Western blots appear to have minimal background details, and both the Western blots and flow cytometry scatter plots share similarities with those present in papers by different authors. The authors were contacted regarding the concerns, however so far the Editorial Office has not received a response.

